# Membranous Dysmenorrhœa

**Published:** 1875-10

**Authors:** A. W. Robinson


Membranous Dysmenorrhœa.—*By A. W. Robinson, M.D.*—In November, 1872, I was consulted by Mrs. J. R., on account of membranous dysmenorrhœa. In view of the futility of my efforts, up to that time, to make radical cures, or render fertile patients of this kind by the treatment recommended, and, having often seen the almost specific power of opium over congestion and inflammation of the other mucous membranes, and having frequently witnessed the palliative effect of a dose, given with a view to relieve pain in membranous dysmenorrhœa, I directed her to begin the use of morphia a couple of days before she expected to menstruate, and continue it during the flow.

The dose given was one-fourth of a grain, and was to be repeated sufficiently often to keep her constantly under its influence, and prevent pain. She reported, "no membrane came away, and the treatment afforded great relief." I continued this treatment eighteen months, and as no membrane or shreds had appeared for some time, I discontinued it.

In November, 1874, she became pregnant, and was delivered August 8, 1875. I do not consider it proved that the treatment and recovery bear the relation of cause and effect. That no deduction can be made from one case, I am well aware, but think this treatment merits a further trial.—*Med. and Surg. Reporter.*

